# Mixed Movement Disorder Caused by *ADCY5* Pathogenic Variant Successfully Treated With Caffeine: A Case From Ukraine

**DOI:** 10.1155/crnm/4661314

**Published:** 2026-03-06

**Authors:** Eugenia Tsoma, Taras Studeniak, Robert Jech, Michael Zech, Petra Havrankova, Evžen Růžička, Lukas Kunc, Yuriy Chomolyak

**Affiliations:** ^1^ Department of Family Medicine and Outpatient Care, Second Faculty of Medicine, Uzhhorod National University, Uzhhorod, Ukraine; ^2^ Department of Neurology and Center of Clinical Neuroscience, First Faculty of Medicine, Charles University and General University Hospital in Prague, Prague, Czech Republic, cuni.cz; ^3^ Department of Neurology, Neurosurgery and Psychiatry, First Faculty of Medicine, Uzhhorod National University and Regional Clinical Center of Neurosurgery and Neurology, Uzhhorod, Ukraine; ^4^ Institute of Neurogenomics, Helmholtz Zentrum München, Munich, Germany, helmholtz-muenchen.de; ^5^ Institute of Human Genetics, Technical University of Munich, Munich, Germany, tum.de; ^6^ Institute for Advanced Study, Technical University of Munich, Garching, Germany, tum.de; ^7^ Diamed Medical Center, Uzhhorod, Ukraine

**Keywords:** caffeine, MxMD-*ADCY5*, paroxysmal dyskinesia

## Abstract

**Background:**

Mixed movement disorder due to pathogenic variants in the *ADCY5* gene (MxMD‐*ADCY5*) is a rare condition characterized predominantly by paroxysmal involuntary choreiform movements involving the limbs, face, and neck, often accompanied by dystonia and myoclonus. Speech disturbances, delayed motor development, cognitive impairment, axial hypotonia, and episodic exacerbations of dyskinesia are also common features. Currently, treatment strategies remain limited; however, several studies indicate a beneficial effect of caffeine in the management of dyskinesia.

**Objective:**

This clinical report aims to present a case of MxMD‐*ADCY5* showing a high therapeutic response to caffeine. To our knowledge, this is the first reported Ukrainian patient with this condition demonstrating dramatic improvement in paroxysmal dyskinesia after treatment with high‐dose caffeine.

**Conclusions:**

In our clinical observation, administration of caffeine at a total daily dose of 600 mg resulted in significant clinical improvement in an adult patient with MxMD*-ADCY5* over a follow‐up period of at least 6 months. No adverse events or side effects were reported.

## 1. Introduction

Mixed movement disorder–adenylyl cyclase isoform 5 (MxMD‐*ADCY5*) is a rare early‐onset neurological condition characterized by involuntary movements primarily affecting the limbs, face, and neck. It is often accompanied by paroxysmal dyskinesia and is frequently misdiagnosed as epileptic seizures or dyskinetic cerebral palsy [1, 2]. Other common clinical features include axial hypotonia and episodic exacerbations of dyskinesia, precipitated by sleep, emotional stress, intercurrent illness, or occurring without identifiable triggers [3, 4]. The movements are often continuous during waking hours or can disrupt sleep [4]. *ADCY5* is part of the adenylyl cyclase (AC) family, a group of enzymes that convert adenosine triphosphate (ATP) into cyclic adenosine‐3′,5′‐monophosphate (cAMP). Because cAMP functions as a key intracellular second messenger, pathogenic variants in genes encoding ACs can disrupt downstream signaling pathways [5, 6]. De novo pathogenic variants and somatic mosaicism are frequently observed in the *ADCY5* gene [3, 6, 7]. Although the condition is typically inherited in an autosomal dominant manner, several cases with recessive inheritance have been described [6, 8, 9]. The disorder shows genetic heterogeneity: most variants lead to a gain‐of‐function effect, but loss‐of‐function pathogenic variants and *ADCY5* haploinsufficiency have also been reported [6, 7].

To the best of our knowledge, no previous cases from Ukraine have been reported. Herein, we describe a Ukrainian patient with MxMD‐*ADCY5* who showed a marked improvement with high‐dose caffeine therapy.

## 2. Clinical Case Description

### 2.1. Patient Information

A 32‐year‐old Ukrainian male with no familial history of similar disorders was born at full term without perinatal complications. Nevertheless, his psychomotor milestones were delayed; he began speaking at the age of five and walking at the age of six. At the age of 10 years, he developed recurrent involuntary movements including eye blinking, mouth opening, and severe trunk and limb choreo‐dystonic movements. The attacks always occurred during bedtime and persisted for hours each night (with up to 15–20 episodes per night, each lasting 20–30 min and separated by brief intervals—see video 1).

Although brain MRI and EEG did not reveal pathologic changes, a diagnosis of dyskinetic cerebral palsy with symptomatic epilepsy was initially made by pediatric neurologists. Numerous pharmacological attempts—including benzodiazepines, valproic acid, carbamazepine, lamotrigine, gabapentin, and pregabalin—had been initiated, but without notable improvement.

### 2.2. Clinical Findings

In September 2024 the patient was referred to an epileptologist for further control video‐electroencephalogram. No medication was used at that time. Still, no pathological epileptiform activity during 24 h monitoring was found, and paroxysmal dyskinesia was suspected.

Neurological examination showed slowing of horizontal saccades, oculomotor apraxia, dysarthria, mild orofacial dyskinesia, distal choreoathetosis of upper limbs with mild myoclonic jerks, mild retrocollis, axial and limb muscles hypotonia, and an ataxic gait. Although these symptoms were constant, they did not cause the patient as much discomfort as the predominance of severe exhausting disabling nocturnal dyskinetic attacks. The remainder of the neurological examination was normal. No intellectual deficiency was found. Periodic aggressive behavior was noted by the patient’s mother. The family history was negative. The basic metabolic workups were normal.

### 2.3. Diagnostic Assessment

Whole exome sequencing (WES) was performed at the Institute of Human Genetics, Technical University of Munich, Germany. In January 2025, a pathogenic autosomal dominant missense variant in the *ADCY5* gene was identified: *NM_183357.3 (ADCY5): c.1252C > T, p.(Arg418Trp).* The variant was not confirmed by a method other than WES. The platform Illumina was used.

### 2.4. Administration of Therapeutic Intervention and Patient’s Follow‐Up

Caffeine at a dose of 200 mg twice daily (preferably in the morning and afternoon) was prescribed as a first‐line therapeutic intervention immediately after obtaining the genetic results. Total resolution of nocturnal dyskinetic attacks was observed after the first day of caffeine usage (see video 2). One week later, dyskinetic episodes slightly re‐emerged, but increasing the dose of caffeine to 600 mg divided into two doses contributed to complete control over nocturnal severe dyskinesias and dramatic improvement of daily living and sleep quality (no nighttime awakenings due to recurrent hyperkinetic episodes). However, caffeine had no significant effect on axial hypotonia, and mild choreiform movements in the neck muscles and distal extremities have persisted.

The effect of caffeine has been stable over the past 6 months since the end of January 2025.

Notably, the patient had not previously consumed caffeine in the form of coffee drinks, as his treating physicians advised against it due to concerns that it might precipitate seizures. Consequently, caffeine was administered in tablet form to ensure precise dose control.

## 3. Discussion

The majority of individuals diagnosed with MxMD‐*ADCY5* carry a de novo pathogenic variant [4, 10–13]. In light of instances of *ADCY5* mosaicism, the ongoing WES analysis of both parents will clarify whether the condition in this patient is inherited or arises sporadically.

Kozon and colleagues, in their systematic review of cases published between January 2017 and January 2022, identified 50 newly described patients with pathogenic *ADCY5* variants across 25 publications and compared them with two cases from their own cohort. They reported that dystonia represents the most frequent clinical manifestation of MxMD‐*ADCY5*, occurring in more than 80% of affected individuals, whereas chorea is observed in approximately two‐thirds of patients [6, 13]. Dyskinesias, speech disturbances, and delayed motor development are also common, each affecting over half of cases. Hypotonia and myoclonus have been described in nearly half and 40% of patients, respectively, and cognitive impairment is reported in approximately one‐fifth of cases [6, 10]. Sleep‐related symptoms constitute an important clinical feature of MxMD‐*ADCY5*. Around one‐third of patients present sleep disturbances, ranging from restless sleep to recurrent nocturnal hyperkinetic episodes or painful exacerbations of dyskinesia during sleep. In several reports, nocturnal symptoms were considerably more severe than daytime manifestations, and episodes of generalized dystonia accompanied by inconsolable crying have been documented [6]. Méneret and colleagues, using video polysomnography, found that abnormal movements commonly precipitate prolonged awakenings, resulting in poor sleep quality characterized by low sleep efficiency and difficulty returning to sleep, rather than problems with sleep initiation. These movements occur most frequently during transitions from sleep to wakefulness (particularly in the morning) as well as during N2 and REM sleep stages, and they resemble movements observed during wakefulness. Sleep onset latency, however, was typically quick [14]. Unfortunately, for technical reasons, it was not possible to perform polysomnography in our case and detail episodes of nocturnal dyskinetic attacks. Almost the full spectrum of extrapyramidal manifestations was observed in our patient, with a predominance of nocturnal dyskinetic episodes but without accompanying cognitive decline.

Currently, no randomized controlled trials or consensus guidelines are available for the treatment of MxMD‐*ADCY5.* Some reports suggest that benzodiazepines, such as clonazepam, clobazam, diazepam, and lorazepam, can help in managing dyskinetic episodes [4, 10, 15]. In a study involving 30 individuals with ADTY5‐related dyskinesia, a beneficial effect of caffeine on hyperkinetic movements was observed. Twenty‐six patients (87%) reported an improvement in overall movement disorders of 40% or more and thus were considered to be caffeine responders. Caffeine in different doses from 60 to 600 mg reduced the frequency and duration of paroxysmal dyskinesias and also improved other motor and nonmotor features that have an impact on quality of life [16].

Our case report describes a dramatic improvement of dyskinetic movements with predominantly nocturnal events, which were totally resolved for more than 6 months from the beginning of caffeine intake of 600 mg daily divided into two equal doses. Improvements were also noted in other clinical characteristics (gait, general mobility, subjective psychosocial status, and quality of sleep) and patient’s quality of life in general. Additionally, no adverse effects were reported by the patient or family members. This effect, as noted, is thought to be attributed to the attenuating action of caffeine as an adenosine A2A antagonist, which is believed to lead to a reduction of cAMP levels in the striato‐pallidal projection neurons [16].

Several reports indicate that theophylline exhibits higher affinity for the adenosine A2A receptor, suggesting that it may be more effective than caffeine in treating MxMD‐*ADCY5* [16, 17]. Tänzler et al., based on results of a retrospective case series study, recommend theophylline administration in patients with MxMD‐*ADCY5* as an equivalent treatment option to caffeine. Blood drug levels should optimally range between 15 and 20 mg/L to decrease dyskinetic movements and to improve overall quality of life [4, 17]. Frequent monitoring of blood theophylline levels is crucial, and therapy must be administered with great caution, as even lower doses can be associated with side effects like tachycardia, vomiting, headaches, nausea, impaired sleep, and agitation, whereas higher doses can lead to significant toxicity, such as seizures, hypotension, and significant arrhythmias [4]. We hope that theophylline may be an additional therapy option for our patient in case the caffeine loses its potency with time.

In recent studies, patients with MxMD‐*ADCY5* may benefit from bilateral pallidal deep brain stimulation (DBS) [18–20]. However, other clinical features, such as axial hypotonia, did not respond to DBS [20]. DBS may be an option for this patient if it is covered by the national health program in the future.

## 4. Summary

This report describes the first Ukrainian patient with MxMD‐*ADCY5*, who presented with a high response to caffeine. We suspect that there may be more such patients with misdiagnoses of paroxysmal dyskinesia in Ukraine and other countries. Therefore, genetic testing (particularly WES) is essential for establishing the diagnosis. Even when genetic testing is unavailable, the presence of childhood‐onset nocturnal paroxysmal dyskinesia—especially when accompanied by persistent orofacial dyskinesia and hypotonia—should prompt consideration of a therapeutic trial of caffeine. Further novel studies are needed to compare the efficacy and tolerability, as well as the optimal dosage of caffeine and/or theophylline or their combination, for patients with MxMD‐*ADCY5*.

**Nomenclature**
ADCY5: Adenylyl cyclase 5
ATP: Adenosine triphosphate
cAMP: Cyclic adenosine monophosphate
DBS: Deep brain stimulation
EEG: Electroencephalography
MxMD‐ADCY5: Mixed movement disorder‐ADCY5
MRI: Magnetic resonance imaging
WES: Whole exome sequencing

## Author Contributions

Eugenia Tsoma: manuscript conception, collection, and analysis of clinical, genetic, and laboratory data, writing of the first draft; Taras Studeniak: analysis of clinical data, manuscript review, and critique; Robert Jech: study conception and organization, manuscript review, and critique; Michael Zech: genetic study and data interpretation, bioinformatics analysis, manuscript review, and critique; Petra Havrankova: study conception and organization, manuscript review, and critique; Evžen Růžička: manuscript review and critique; Lukas Kunc: clinical data collection, manuscript review, and critique; Yuriy Chomolyak: clinical data collection, manuscript review, and critique.

## Funding

The work was supported by the Agency for Health Research of the Czech Republic (grant AZV:NW24‐04‐00067) and also by the Charles University: Cooperation Program in Neuroscience.

It was also supported by a “*Schlüsselprojekt*” grant from the Else Kröner‐Fresenius‐Stiftung (2022_EKSE.185 to M.Z.). Our work was also supported by funding from the EJP RD (EJP RD Joint Transnational Call 2022) and the German Federal Ministry of Education and Research (BMBF, Bonn, Germany), awarded to the project PreDYT (PREdictive biomarkers in DYsTonia, 01GM2302). In addition, this study (M.Z.) has received funding from the Federal Ministry of Education and Research (BMBF) and the Free State of Bavaria under the Excellence Strategy of the Federal Government and the Länder, as well as by the Technical University of Munich‐Institute for Advanced Study.

## Disclosure

*Financial Disclosures for the previous 12 months:* The authors declare that there are no additional disclosures to report.

## Consent

Written informed consent was obtained from the participant for the publication of this report.

## Conflicts of Interest

The authors declare no conflicts of interest.

## Supporting Information

Additional supporting information can be found online in the Supporting Information section.

Video Legend: Patient with ADCY5‐related dyskinesia during bedtime before and a day after caffeine prescription.

## Supporting information


**Supporting Information 1** Video 1. Severe night‐time dyskinetic attack before the treatment with caffeine started.

**Supporting Information 2** Video 2. Marked reduction of severe nocturnal dyskinetic episodes noted one day after initiation of caffeine therapy, with only mild residual hyperkinetic movements of the face and upper limbs, accompanied by axial hypotonia.
